# SARS-CoV-2 antibody prevalence by industry, workplace characteristics, and workplace infection prevention and control measures, North Carolina, USA, 2021 to 2022

**DOI:** 10.1093/annweh/wxae067

**Published:** 2024-08-05

**Authors:** Carolyn Gigot, Nora Pisanic, Kristoffer Spicer, Meghan F Davis, Kate Kruczynski, Magdielis Gregory Rivera, Kirsten Koehler, D J Hall, Devon J Hall, Christopher D Heaney

**Affiliations:** Department of Environmental Health and Engineering, Johns Hopkins Bloomberg School of Public Health, Baltimore, MD, United States; Department of Environmental Health and Engineering, Johns Hopkins Bloomberg School of Public Health, Baltimore, MD, United States; Department of Environmental Health and Engineering, Johns Hopkins Bloomberg School of Public Health, Baltimore, MD, United States; Department of Environmental Health and Engineering, Johns Hopkins Bloomberg School of Public Health, Baltimore, MD, United States; Johns Hopkins P.O.E. Total Worker Health(R) Center in Mental Health, Baltimore, MD, United States; Division of Infectious Diseases and Department of Molecular and Comparative Pathobiology, Johns Hopkins School of Medicine, Baltimore, MD, United States; Department of Environmental Health and Engineering, Johns Hopkins Bloomberg School of Public Health, Baltimore, MD, United States; Department of Environmental Health and Engineering, Johns Hopkins Bloomberg School of Public Health, Baltimore, MD, United States; Department of Environmental Health and Engineering, Johns Hopkins Bloomberg School of Public Health, Baltimore, MD, United States; Rural Empowerment Association for Community Help, Warsaw, NC, United States; Rural Empowerment Association for Community Help, Warsaw, NC, United States; Department of Environmental Health and Engineering, Johns Hopkins Bloomberg School of Public Health, Baltimore, MD, United States; Department of Epidemiology, Johns Hopkins Bloomberg School of Public Health, Baltimore, MD, United States; Department of International Health, Johns Hopkins Bloomberg School of Public Health, Baltimore, MD, United States; Community Science and Innovation for Environmental Justice Initiative, Center for a Livable Future, Department of Environmental Health and Engineering, Johns Hopkins Bloomberg School of Public Health, Baltimore, MD, United States

**Keywords:** animal slaughtering and processing, COVID-19, industrial livestock operations, seroprevalence

## Abstract

The COVID-19 pandemic has disproportionately affected workers in certain industries and occupations, and the workplace can be a high-risk setting for SARS-CoV-2 transmission. In this study, we measured SARS-CoV-2 antibody prevalence and identified work-related risk factors in a population primarily working at industrial livestock operations. We used a multiplex salivary SARS-CoV-2 IgG assay to determine infection-induced antibody prevalence among 236 adult (≥18 yr) North Carolina residents between February 2021 and August 2022. We used the National Institute for Occupational Safety and Health Industry and Occupation Computerized Coding System (NIOCCS) to classify employed participants’ industry. Most participants (55%, 95% confidence interval [CI] 49% to 62%) were infection-induced IgG positive, including 71% (95% CI 60% to 83%) of animal slaughtering and processing industry workers, 1.5 to 4.3 times North Carolina general population infection-induced seroprevalence estimates during overlapping time periods. Considering self-reported diagnostic test positivity and vaccination history in addition to antibodies, the proportion of participants with evidence of prior infection increased slightly to 61% (95% CI 55% to 67%), including 75% (95% CI 64% to 87%) of animal slaughtering and processing workers. Participants with more than 1000 compared to 10 or fewer coworkers at their jobsite had higher odds of prior infection (adjusted odds ratio 4.5, 95% CI 1.0 to 21.0). This study contributes evidence of the severe and disproportionate impacts of COVID-19 on animal slaughtering and processing workers and workers in large congregate settings.

**What’s Important About This Paper?**

This study observed high salivary SARS-CoV-2 infection-induced IgG prevalence in animal slaughtering and processing industry workers (71%) between February 2021 and August 2022 in North Carolina, USA. Higher odds of SARS-CoV-2 infection among participants were observed at worksites with larger compared to smaller numbers of employees. This study adds to the evidence of high SARS-CoV-2 transmission among livestock industry workers in the United States.

## Introduction

COVID-19 continues to impact numerous risk groups, including workers. COVID-19 outbreaks at United States meat and poultry processing operations and long-term care facilities were reported early in spring 2020, and more than 12,000 workplace COVID-19 outbreaks were reported by 23 health departments during August to October 2021 (Luckhaupt et al. 2023). More than 59,000 worker COVID-19 cases were reported by 5 US meat processing companies during the first year of the pandemic in the US (House Staff Memorandum 2021). Studies have found higher COVID-19 mortality among particular occupational sectors, including farming, construction, production, transportation, and healthcare (Hawkins et al. 2021; Billock et al. 2022; Cummings et al. 2022).

The disproportionality of COVID-19 cases and deaths is connected to many interrelated factors. Co-morbidities, co-exposures, socioeconomic status, and limited access to paid sick leave or healthcare can increase workers’ disease susceptibility and worsen outcomes (Carlsten et al. 2021). Workplace characteristics and job tasks, including prolonged close contact with coworkers, clients, customers, or patients; insufficient ventilation; and lack of appropriate personal protective equipment (PPE) can increase workers’ exposures to SARS-CoV-2 (Carlsten et al. 2021). Research has associated workplace SARS-CoV-2 infection prevention and control (IPC) measures—including surveillance, improved sick leave practices, improved ventilation, crowding reduction practices, provision of adequate PPE, and universal masking requirements—with reductions in COVID-19 cases (Ingram et al. 2021).

SARS-CoV-2 antibody data can provide information on the cumulative incidence of infection and is not affected by limited access to diagnostic testing or limited reporting of at-home rapid SARS-CoV-2 antigen test results (Pisanic et al. 2020, 2023; Duarte et al. 2022). SARS-CoV-2 seroprevalence surveys have identified higher-risk occupational groups, including manufacturing, meatpacking, farm, and healthcare workers (Klein et al. 2022; Raja et al. 2022; Boucher et al. 2023; Chen et al. 2023; Graham et al. 2023). Meza et al. used serology and infection and vaccination history to identify higher SARS-CoV-2 infection risk for in-person workers and certain occupational groups, including farming, fishing, and forestry, in California during summer 2021; however, their sample did not allow for quantification of infection risk among meatpacking or livestock workers (Meza et al. 2023).

In this study, we used a multiplex salivary SARS-CoV-2 IgG assay to determine infection-induced antibody prevalence in a North Carolina study population primarily working at industrial livestock operations and compared infection by workplace characteristics, including industry and IPC measures.

## Methods

This study was designed and conducted in a collaboration between the Johns Hopkins Bloomberg School of Public Health (BSPH) and the Rural Empowerment Association for Community Help (REACH), a community organization based in Duplin County, North Carolina (Gigot et al. 2023). We recruited households in North Carolina using a snowball sampling approach beginning from REACH organizers’ social networks. The study was designed to enroll participants into 3 groups: participants living in households with at least one adult working at an industrial hog or poultry operation, meatpacking plant, or animal rendering plant (industrial livestock operation [ILO] household group), participants living near these facilities without any known occupational exposure to livestock (ILO neighbors), and participants living in metropolitan areas of North Carolina (metro). Eligibility criteria included at least one adult (≥18) household member participating in the study and the ability to understand spoken English or Spanish. Participants completed a baseline questionnaire and collected a saliva sample using the ORACOL+ Saliva Collection Device (Malvern Medical Developments, Worcester, UK). Saliva samples were tested for SARS-CoV-2 IgG antibodies with an in-house multiplex bead-based assay using Luminex technology which has been described previously (infection-induced nucleocapsid [N] and spike [S] sensitivity = 97.6% and specificity = 99.4%; infection- and/or vaccination-induced [S] sensitivity, 99.4%; specificity, 99.3%) (Heaney et al. 2021; Gigot et al. 2023; Pisanic et al. 2023). The BSPH Institutional Review Board (IRB) approved this study (IRB00014420).

We used the National Institute for Occupational Safety and Health (NIOSH) Industry and Occupation Computerized Coding System (NIOCCS) to identify employed participants’ industry from free text job title, job category, and industrial livestock operation (ILO) category (CDC 2022a). NIOCCS provides detailed 2017 North American Industry Classification System (NAICS) codes, grouped into 21 major sectors. Subsectors with 10 participants or fewer were grouped by major sector; major sectors with 10 participants or fewer were combined into an “Other” category.

We tested for differences in demographic characteristics and COVID-19 outcomes between industry groups using the Kruskal–Wallis rank sum test for continuous variables; Pearson’s Chi-squared test for categorical variables with all expected cell counts ≥5; and Fisher’s exact test for categorical variables with any expected cell count <5. We used binomial regression with a logit link to calculate crude and adjusted odds ratios and 95% confidence intervals (95% CIs) comparing evidence of prior infection with SARS-CoV-2 by workplace characteristics. We used multiple imputations with chained equations to account for missing values in multivariable analyses (<1% missing overall). All statistical analyses were completed in R 4.2.2 (R Core Team 2022).

## Results

Most participants (55% overall) had infection-induced SARS-CoV-2 antibodies, including 71% of animal processing, 62% of animal production, 40% of health care and social assistance, 50% of other industry workers, and 51% of adult participants who were not employed (Table 1). After combining antibody with self-reported viral diagnostic test and vaccination history, more participants (61%) had evidence of prior SARS-CoV-2 infection, including 77% in animal production, 75% in animal processing, 40% in health care and social assistance, 53% in other industries, and 61% of adult participants who were not employed (Supplementary Fig. S1). Animal processing workers had somewhat higher odds of prior SARS-CoV-2 infection compared with animal production, health care, and social assistance, and other industry workers combined, but this association was attenuated after adjustment for sampling date (Supplementary Table S1).

**Table 1.** Adult (≥18) participant (*N* = 236) characteristics by industry, North Carolina, 2021-2022.

Characteristic	Animal slaughtering & processing, *N* = 57	Health care & social assistance, *N* = 20	Animal production & aquaculture, *N* = 13	Other, *N* = 77	Not employed, *N* = 69	*P*-value for any difference
Age in years, median (IQR)	36 (28, 48)	38 (27, 49)	46 (34, 59)	36 (24, 48)	58 (36, 68)	<0.001^a^
Sex, *n* (%)						
Female	32 (56%)	18 (90%)	2 (15%)	50 (65%)	41 (59%)	<0.001^b^
Male	25 (44%)	2 (10%)	11 (85%)	27 (35%)	28 (41%)	
Race/ethnicity, *n* (%)						0.6^c^
Black	51 (89%)	16 (80%)	12 (92%)	63 (83%)	62 (90%)	
Hispanic/Latino	4 (7.0%)	2 (10%)	1 (7.7%)	9 (12%)	5 (7.2%)	
White	1 (1.8%)	2 (10%)	0 (0%)	1 (1.3%)	2 (2.9%)	
Other	1 (1.8%)	0 (0%)	0 (0%)	3 (3.9%)	0 (0%)	
Education, *n* (%)						0.001^c^
<High school	9 (16%)	0 (0%)	3 (23%)	9 (12%)	10 (15%)	
High school	33 (58%)	7 (37%)	6 (46%)	22 (29%)	34 (50%)	
Trade school	1 (1.8%)	0 (0%)	0 (0%)	4 (5.2%)	3 (4.4%)	
Some college	10 (18%)	3 (16%)	4 (31%)	14 (18%)	8 (12%)	
College	4 (7.0%)	7 (37%)	0 (0%)	27 (35%)	11 (16%)	
Post-college	0 (0%)	2 (11%)	0 (0%)	1 (1.3%)	2 (2.9%)	
Household occupants, n (%)						0.003^c^
Only self	11 (19%)	5 (25%)	6 (50%)	23 (30%)	34 (50%)	
1–2 cohabitants	28 (49%)	9 (45%)	5 (42%)	41 (54%)	29 (43%)	
>2 cohabitants	18 (32%)	6 (30%)	1 (8.3%)	12 (16%)	5 (7.4%)	
Uninsured, *n* (%)	4 (7.0%)	1 (5.0%)	1 (7.7%)	11 (14%)	9 (13%)	0.6^c^
Days from WHO pandemic declaration (March 11, 2020) at enrollment, median (IQR)	721 (558, 796)	417 (376, 466)	700 (699, 800)	456 (379, 743)	539 (378, 730)	<0.001^a^
*COVID-19 outcomes*						
*Multiplex IgG antibody assay results*						
SARS-CoV-2 infection-induced IgG (positive for both N and S), *n* (%)	40 (71%)	8 (40%)	8 (62%)	37 (50%)	35 (51%)	0.06^b^
SARS-CoV-2 infection- and/or vaccination-induced IgG (positive for S), *n* (%)	46 (82%)	13 (65%)	11 (85%)	51 (69%)	57 (84%)	0.1^c^
*Questionnaire responses*						
At least one symptom of COVID-19, *n* (%)	38 (67%)	11 (55%)	5 (38%)	45 (58%)	33 (48%)	0.2 ^b^
At least 2 symptoms of COVID-19, *n* (%)	30 (53%)	8 (40%)	5 (38%)	39 (51%)	25 (36%)	0.3 ^b^
Ever thought you had COVID-19, *n* (%)	12 (21%)	5 (25%)	3 (25%)	20 (28%)	9 (14%)	0.3 ^c^
Ever took a viral SARS-CoV-2 test, *n* (%)	34 (61%)	15 (75%)	5 (38%)	50 (65%)	36 (53%)	0.2^b^
Ever positive viral SARS-CoV-2 test, *n* (%)	9 (26%)	3 (20%)	1 (20%)	17 (34%)	15 (42%)	0.5^c^
Ever positive viral SARS-CoV-2 test and no symptoms of COVID-19, *n* (%)	1 (1.8%)	0 (0%)	1 (7.7%)	2 (2.6%)	0 (0%)	0.2^c^
At least one vaccine dose, *n* (%)	30 (53%)	11 (58%)	7 (58%)	36 (49%)	39 (57%)	0.9^b^
Primary vaccine series complete, *n* (%)	25 (45%)	7 (37%)	6 (55%)	30 (42%)	34 (50%)	0.8^c^
*Synthesis of multiplex IgG antibody assay results and reported viral test and vaccination history*						
Evidence of prior SARS-CoV-2 infection, *n* (%)	43 (75%)	8 (40%)	10 (77%)	41 (53%)	42 (61%)	0.02^b^
Evidence of prior SARS-CoV-2 infection and no symptoms of COVID-19, *n* (%)	16 (28%)	3 (15%)	6 (46%)	17 (22%)	22 (32%)	0.2^c^

^a^Kruskal–Wallis rank sum test for any difference in characteristic among industry categories.

^b^Pearson’s Chi-squared test for any difference in characteristic among industry categories.

^c^Fisher’s exact test for any difference in characteristic among industry categories.

*Note:* IQR = interquartile range. See Supplementary Fig. S1 for conceptual decision tree for determining evidence of prior SARS-CoV-2 infection using antibody and reported viral test and vaccination history. Eleven participants were missing information on vaccine series completion and ever thought had COVID-19, 4 on SARS-CoV-2 N and S IgG, 5 on at least one vaccine dose, 3 on age and cohabitants, 2 on education and viral SARS-CoV-2 test history, and 1 on race/ethnicity.

After adjustment for sampling date and industry category, participants with more than 1,000 employees at their worksite had higher odds of prior SARS-CoV-2 infection compared to those with 10 or fewer (aOR 4.5, 95% CI 1.0 to 21.0) (Table 2, Supplementary Table S2).

**Table 2.** Odds of prior SARS-CoV-2 infection by workplace characteristics and infection prevention and control measures among adult (≥18) employed participants (*N* = 167), North Carolina, 2021–2022.

Characteristic	Prior infection/total, *n* (%)	OR (95% CI)	aOR (95% CI) (adjusted for sampling date and industry)
Essential worker			
No	20/41 (49)	-	-
Yes	80/124 (65)	1.9 (0.9, 3.9)	2.2 (0.8, 5.7)
Worked in person past 2 wk (at all)			
No	8/15 (53)	-	-
Yes	94/152 (62)	1.4 (0.5, 4.2)	0.9 (0.3, 3.0)
Employees at worksite			
10 or fewer (reference)	21/43 (49)	-	-
11–100	32/48 (67)	2.1 (0.9, 4.9)	2.1 (0.8, 5.5)
101–1,000	24/43 (56)	1.3 (0.6, 3.1)	1.0 (0.4, 2.8)
>1,000	22/25 (88)	7.7 (2.0, 29.8)	4.5 (1.0, 21.0)
Hours worked per week			
<40	19/35 (54)	-	-
40	31/52 (60)	1.2 (0.5, 3.0)	1.4 (0.5, 3.9)
>40	52/80 (65)	1.6 (0.7, 3.5)	1.3 (0.5, 3.3)
Aware of COVID-19 cases at work past 2 wk			
No	12/23 (52)	-	-
Yes	89/143 (62)	1.5 (0.6, 3.7)	2.1 (0.8, 5.6)
Able to maintain 6+ feet of distance			
No	21/39 (54)	-	-
Yes	80/127 (63)	1.5 (0.7, 3.0)	1.2 (0.5, 2.8)
Could isolate if COVID-19+ without losing job	89/151 (59)	-	-
Could not	9/10 (90)	6.3 (0.8, 51.6)	4.1 (0.5, 37.3)
Could quarantine if COVID-19 exposed without losing job	90/152 (59)	-	-
Could not	8/9 (89)	5.5 (0.7, 46.0)	3.4 (0.4, 31.3)
*Infection prevention and control measures*			
*Engineering controls*			
Physical barriers between stations			
No	69/113 (61)	-	-
Yes	33/54 (61)	1.0 (0.5, 2.0)	1.1 (0.5, 2.2)
Added hand washing stations			
No	31/53 (58)	-	-
Yes	71/114 (62)	1.2 (0.6, 2.3)	1.4 (0.6, 3.0)
*Administrative controls*			
Change in workplace sick leave or bonus program policies			
No	77/119 (65)	-	-
Yes	25/48 (52)	0.6 (0.3, 1.2)	1.0 (0.5, 2.2)
COVID-19 testing at work			
No	66/109 (61)	-	-
Yes	36/58 (62)	1.1 (0.6, 2.1)	0.9 (0.4, 2.0)
*PPE*			
Masks required			
No	24/37 (65)	-	-
Yes	78/130 (60)	0.8 (0.4, 1.7)	1.2 (0.5, 2.9)
Employer provides face masks (any type)			
No	17/26 (65)	-	-
Yes	85/141 (60)	0.8 (0.3, 1.9)	0.9 (0.3, 2.5)
Employer provides N95/KN95/respirators			
No	81/132 (61)	-	-
Yes	21/35 (60)	0.9 (0.4, 2.0)	0.7 (0.3, 1.8)
Employer provides surgical masks			
No	43/67 (64)	-	-
Yes	59/100 (59)	0.8 (0.4, 1.5)	1.1 (0.5, 2.3)
Employer provides cloth masks			
No	76/126 (60)	-	-
Yes	26/41 (63)	1.1 (0.5, 2.4)	1.0 (0.4, 2.3)
Employer provides face shields			
No	68/115 (59)	-	-
Yes	34/52 (65)	1.3 (0.7, 2.6)	1.1 (0.5, 2.4)
Employer provides gloves			
No	46/81 (57)	-	-
Yes	56/86 (65)	1.4 (0.8, 2.7)	1.1 (0.5, 2.4)

*Note:* OR = odds ratio; CI = confidence interval; aOR = adjusted odds ratio. Sampling date was modeled as continuous (days from WHO COVID-19 pandemic declaration, March 11, 2020 (Ghebreyesus, 2020), to sampling date); industry was modeled as categorical (animal slaughtering and processing, health care and social assistance, animal production and aquaculture, other). Nine participants were missing information on number of employees at worksite; 8 on ability to isolate/quarantine; 3 on essential worker status; 2 on awareness of COVID-19 cases at work; and 1 on hours worked per week.

## Discussion

The prevalence of SARS-CoV-2 infection-induced IgG prevalence among adults in our study population was 55%, higher than North Carolina general population infection-induced seroprevalence estimates during overlapping periods (18% to 52%) (Table 1, Supplementary Tables S3 and S4, Supplementary Fig. S2) (CDC 2020a, 2020b). Animal processing industry workers had the highest SARS-CoV-2 infection-induced IgG prevalence: 71%, 1.4 to 3.9-fold higher than North Carolina general population estimates (CDC 2020a, 2020b). Animal processing industry workers’ seroprevalence (between February 2021 and August 2022) was also 8.4 times that of the North Carolina Cabarrus County COVID-19 Prevalence and Immunity Study cohort sampled in November 2021 (8.5%) and 3.1 times that of the COVID-19 Prevention in Emory Healthcare Personnel cohort, another high-risk worker group tested with the same assay, in January to December 2021 (23.2%) (Neighbors et al. 2022; Gigot et al. 2023). We found similar seroprevalence compared to another animal slaughtering and processing industry worker seroprevalence study in fall 2020 (Klein et al. 2022, Supplementary Table S5). While our snowball sampling approach and different sampling periods complicate these comparisons, our results suggest continued transmission and elevated risk among animal slaughtering and processing workers during 2021 and 2022.

Participants with more than 1,000 employees at their worksite had higher adjusted odds of SARS-CoV-2 infection compared to participants with 10 or fewer (Table 2), potentially driven by increased contact with infected coworkers. Close contact with 10 or more coworkers has been associated with reported COVID-19 exposure at work among workers diagnosed with COVID-19 (Free et al. 2022). Many worksites with more than 1,000 employees in this study were large pork processing facilities, with many other factors contributing to SARS-CoV-2 transmission risk—close proximity, long shifts, cold temperatures, low humidity, poor ventilation, and high levels of noise necessitating yelling to communicate (Taylor et al. 2020; Waltenburg et al. 2020; Klein et al. 2022).

Comprehensive COVID-19 IPC measures (contact tracing and case isolation, facilitating smaller cohorts, viral testing, masking) have been associated with lower COVID-19 positivity in a variety of workplace settings (Ingram et al. 2021). Universal masking and physical barriers have been associated with reductions in COVID-19 incidence in meat processing facilities (Herstein et al. 2021). We did not find associations between any reported IPC measures and adjusted odds of SARS-CoV-2 infection, potentially related to our small sample size, changes over time, differences between industries, limited IPC questions, other factors with larger effects on COVID-19 risk, and/or unmeasured or residual confounding.

This analysis has several limitations. Participants were recruited via convenience and snowball sampling and differ from the general employed North Carolina population. Participants might have been more interested in participating in a COVID-19 study given greater concern around the pandemic, at work or more generally. Given the cross-sectional nature of our analysis, we were not able to establish the timing of industry employment and workplace characteristics before versus after SARS-CoV-2 infection. Some of the higher prevalence of evidence of prior SARS-CoV-2 infection among animal processing compared to other workers in our study is likely due to the generally later enrollment of animal processing workers and the generally increasing prevalence of evidence of infection over the course of the COVID-19 pandemic (Table 1, Supplementary Table S1). We sought to control for covariates related to both work and COVID-19 risk but cannot rule out unmeasured and residual confounding. Our study period (February 2021 to August 2022) included periods of dynamic rates of transmission of COVID-19 and changing public and occupational health guidance (CDC 2020a, 2022b); our limited sample size prevented stratification by shorter time periods. We also did not ask about all COVID-19 infection prevention and control measures of interest, including employer support or requirements for worker vaccination. Our limited sample size prevented analysis by more detailed industry or occupational groups, which might have resulted in exposure misclassification or missed associations in smaller industry sectors or subsectors, and which is an important consideration for future work.

## Conclusions

This study adds to evidence of the severe, disproportionate effects of COVID-19 among livestock industry workers. Animal slaughtering and processing industry workers had higher rates of SARS-CoV-2 infection-induced antibody positivity compared with North Carolina general population estimates during overlapping periods. Workers with more coworkers at their jobsite also had higher odds of evidence of prior SARS-CoV-2 infection compared to workers with fewer, adding to evidence of higher infection risk in large, congregate workplaces.

## Supplementary material

Supplementary material is available at *Annals of Work Exposures and Health* online.

wxae067_suppl_Supplementary_Material

## Data Availability

Deidentified data and code can be requested by email (cg3525@cumc.columbia.edu and cheaney1@jhu.edu).
