# Supplementary material for: SARS-CoV-2 antibody prevalence by industry, workplace characteristics, and workplace infection prevention and control measures, North Carolina, USA, 2021 to 2022
Source: Ann Work Expo Health. 2024 Aug 5;68(8):881–9. doi: 10.1093/annweh/wxae067 (PMC11427537; doi:10.1093/annweh/wxae067)
Supplement: wxae067_suppl_Supplementary_Material [file wxae067_suppl_supplementary_material.pdf]

**SARS-CoV-2 antibody prevalence by industry, workplace characteristics, and workplace infection prevention and control measures, North Carolina, USA, 2021 to 2022**

Carolyn Gigot,[a#*] Nora Pisanic,[a] Kristoffer Spicer,[a] Meghan F. Davis,[abc] Kate Kruczynski,[a**] Magdielis Gregory Rivera,[a***] Kirsten Koehler,[a] D. J. Hall, Jr.,[d] Devon J. Hall,[d] Christopher D. Heaney [aefg#]

[a]Department of Environmental Health and Engineering, Johns Hopkins Bloomberg School of Public Health, Baltimore, Maryland, USA

[b]Johns Hopkins P.O.E. Total Worker Health(R) Center in Mental Health, Baltimore, Maryland, USA

[c]Division of Infectious Diseases and Department of Molecular and Comparative Pathobiology, Johns Hopkins University School of Medicine, Baltimore, Maryland, USA

[d]Rural Empowerment Association for Community Help, Warsaw, North Carolina, USA

[e]Department of Epidemiology, Johns Hopkins Bloomberg School of Public Health, Baltimore, Maryland, USA

[f]Department of International Health Johns Hopkins Bloomberg School of Public Health, Baltimore, Maryland, USA

[g]Community Science and Innovation for Environmental Justice Initiative, Center for a Livable Future, Department of Environmental Health and Engineering, Johns Hopkins Bloomberg School of Public Health, Baltimore, Maryland, USA

#Address correspondence to Carolyn Gigot, cg3525@cumc.columbia.edu, and Christopher D. Heaney, cheaney1@jhu.edu

*Present address: Carolyn Gigot, Department of Environmental Health Sciences, Columbia University Mailman School of Public Health, New York, New York, USA

**Present address: Kate Kruczynski, Maryland Department of Health, Baltimore, Maryland, USA

***Present address: Magdielis Gregory Rivera, Amentum, Falls Church, Virginia, USA

***Figure S1.*** *Conceptual decision tree for determining evidence of prior SARS-Cov-2 infection (green boxes) or no evidence of prior infection (grey boxes) using antibody (anti-N and anti-S antibodies) and questionnaire data (ever tested positive on a viral test, ever received any COVID-19 vaccine), based on Duarte et al., 2022.*

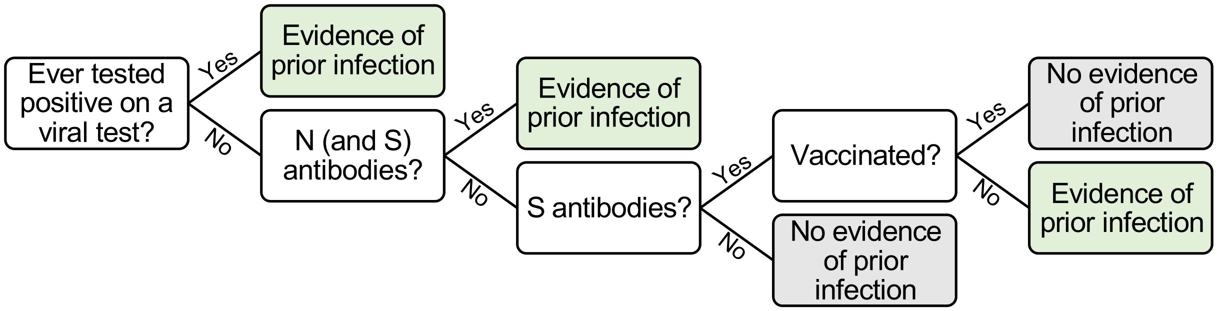


*Note:* Infection with the virus elicits both anti-N and anti-S antibodies. The algorithm for infection-induced antibody positivity including both S and N antigens performed better (higher specificity) compared to the algorithm using N only (Pisanic *et al.*, 2023).

***Table S1.*** *Odds of prior SARS-CoV-2 infection among adult (≥18) employed participants in each industry sector compared to those in all others (N=167), North Carolina, 2021-2022.*

| **Industry sector** | **Prior infection/total, n (%)** | **Reference (employed in all other industry sectors)** | **OR (95% CI)** | **aOR (95% CI) (sampling date)** | **aOR (95% CI) (sampling date and household occupants)** | **aOR (95% CI) (sampling date, household occupants, age, and sex)** |
|---|---|---|---|---|---|---|
| Animal production & aquaculture | 10/13 (77) | 92/154 (60) | 2.2 (0.6, 8.6) | 1.2 (0.3, 4.9) | 1.3 (0.3, 5.5) | 1 (0.2, 4.6) |
| Animal slaughtering & processing | 43/57 (75) | 59/110 (54) | 2.7 (1.3, 5.4) | 1.6 (0.7, 3.5) | 1.5 (0.6, 3.3) | 1.5 (0.7, 3.6) |
| Other | 41/77 (53) | 61/90 (68) | 0.6 (0.3, 1.1) | 0.9 (0.5, 1.9) | 1 (0.5, 2.1) | 1 (0.5, 2.2) |
| Health care & social assistance | 8/20 (40) | 94/147 (64) | 0.4 (0.1, 1.0) | 0.6 (0.2, 1.8) | 0.5 (0.2, 1.6) | 0.5 (0.2, 1.6) |

*Note:* OR = odds ratio; CI = confidence interval; aOR = adjusted odds ratio. Sampling date was modelled as continuous (days from WHO COVID-19 pandemic declaration, March 11, 2020 (Ghebreyesus, 2020), to sampling date); number of household occupants was modelled as categorical (live alone, live with 1-2 cohabitants, live with >2 cohabitants); age was modelled as categorical (30 or younger, 31 to 40, 41 to 50, 51 to 60, 61 or older); sex was modelled as binary (female or male).

***Table S2.*** *Odds of prior SARS-CoV-2 infection by workplace characteristics and infection prevention and control measures among adult (≥18) employed participants (N=167), North Carolina, 2021-2022.*

| **Characteristic** | **Prior infection/total, n (%)** | **OR (95% CI)** | **aOR (95% CI) (sampling date)** | **aOR (95% CI) (sampling date and industry)** | **aOR (95% CI) (sampling date, industry, age, and sex)** |
|---|---|---|---|---|---|
| Essential worker | | | | | |
| No | 20/41 (49) | - | - | - | - |
| Yes | 80/124 (65) | 1.9 (0.9, 3.9) | 2.2 (1, 4.8) | 2.2 (0.8, 5.7) | 2.2 (1, 4.8) |
| Worked in person past 2 weeks (at all) | | | | | |
| No | 8/15 (53) | - | - | - | - |
| Yes | 94/152 (62) | 1.4 (0.5, 4.2) | 1.1 (0.3, 3.3) | 0.9 (0.3, 3.0) | 0.8 (0.2, 2.7) |
| Employees at worksite | | | | | |
| 10 or fewer (reference) | 21/43 (49) | - | - | - | - |
| 11-100 | 32/48 (67) | 2.1 (0.9, 4.9) | 1.9 (0.7, 4.7) | 2.1 (0.8, 5.5) | 2.1 (0.8, 5.6) |
| 101-1000 | 24/43 (56) | 1.3 (0.6, 3.1) | 1.1 (0.4, 2.7) | 1.0 (0.4, 2.8) | 1.1 (0.4, 2.9) |
| >1000 | 22/25 (88) | 7.7 (2.0, 29.8) | 4.6 (1.1, 18.9) | 4.5 (1.0, 21.0) | 5.2 (1.1, 25) |
| Hours worked per week | | | | | |
| <40 | 19/35 (54) | - | - | - | - |
| 40 | 31/52 (60) | 1.2 (0.5, 3.0) | 1.4 (0.5, 3.7) | 1.4 (0.5, 3.9) | 1.4 (0.5, 4) |
| >40 | 52/80 (65) | 1.6 (0.7, 3.5) | 1.5 (0.6, 3.7) | 1.3 (0.5, 3.3) | 1.3 (0.5, 3.4) |
| Aware of COVID-19 cases at work past 2 weeks | | | | | |
| No | 12/23 (52) | - | - | - | - |
| Yes | 89/143 (62) | 1.5 (0.6, 3.7) | 2.1 (0.8, 5.5) | 2.1 (0.8, 5.6) | 1.9 (0.7, 5.4) |
| Able to maintain 6+ feet of distance | | | | | |
| No | 21/39 (54) | - | - | - | - |
| Yes | 80/127 (63) | 1.5 (0.7, 3.0) | 1.2 (0.6, 2.7) | 1.2 (0.5, 2.8) | 1.3 (0.6, 2.9) |
| Could isolate if COVID-19+ without losing job | 89/151 (59) | - | - | - | - |
| Could not | 9/10 (90) | 6.3 (0.8, 51.6) | 3.7 (0.4, 32.6) | 4.1 (0.5, 37.3) | 4.6 (0.5, 43.2) |
| Could quarantine if COVID-19 exposed without losing job | 90/152 (59) | - | - | - | - |
| Could not | 8/9 (89) | 5.5 (0.7, 46.0) | 3.5 (0.4, 30.8) | 3.4 (0.4, 31.3) | 3.5 (0.4, 33.3) |
| | | | | | |
| ***Infection prevention and control measures*** | | | | | |
| *Engineering controls* | | | | | |
| Physical barriers between stations | | | | | |
| No | 69/113 (61) | - | - | - | - |
| Yes | 33/54 (61) | 1.0 (0.5, 2.0) | 1 (0.5, 2.1) | 1.1 (0.5, 2.2) | 1.1 (0.5, 2.4) |
| Added hand washing stations | | | | | |

| | | | | | |
|---|---|---|---|---|---|
| No | 31/53 (58) | - | - | - | - |
| Yes | 71/114 (62) | 1.2 (0.6, 2.3) | 1.5 (0.7, 3.2) | 1.4 (0.6, 3.0) | 1.6 (0.7, 3.5) |
| *Administrative controls* | | | | | |
| Change in workplace sick leave or bonus program policies | | | | | |
| No | 77/119 (65) | - | - | - | - |
| Yes | 25/48 (52) | 0.6 (0.3, 1.2) | 0.9 (0.4, 1.9) | 1.0 (0.5, 2.2) | 1.1 (0.5, 2.4) |
| COVID-19 testing at work | | | | | |
| No | 66/109 (61) | - | - | - | - |
| Yes | 36/58 (62) | 1.1 (0.6, 2.1) | 1 (0.5, 2) | 0.9 (0.4, 2.0) | 0.9 (0.4, 2) |
| *PPE* | | | | | |
| Masks required | | | | | |
| No | 24/37 (65) | - | - | - | - |
| Yes | 78/130 (60) | 0.8 (0.4, 1.7) | 1.3 (0.5, 2.9) | 1.2 (0.5, 2.9) | 1.3 (0.5, 3.2) |
| Employer provides face masks (any type) | | | | | |
| No | 17/26 (65) | - | - | - | - |
| Yes | 85/141 (60) | 0.8 (0.3, 1.9) | 0.8 (0.3, 1.9) | 0.9 (0.3, 2.5) | 0.9 (0.3, 2.4) |
| N95/KN95/respirator | | | | | |
| No | 81/132 (61) | - | - | - | - |
| Yes | 21/35 (60) | 0.9 (0.4, 2.0) | 0.7 (0.3, 1.6) | 0.7 (0.3, 1.8) | 0.8 (0.3, 1.8) |
| Surgical masks | | | | | |
| No | 43/67 (64) | - | - | - | - |
| Yes | 59/100 (59) | 0.8 (0.4, 1.5) | 1.1 (0.5, 2.3) | 1.1 (0.5, 2.3) | 1.1 (0.5, 2.5) |
| Cloth masks | | | | | |
| No | 76/126 (60) | - | - | - | - |
| Yes | 26/41 (63) | 1.1 (0.5, 2.4) | 0.9 (0.4, 2.1) | 1.0 (0.4, 2.3) | 1 (0.4, 2.4) |
| Employer provides face shields | | | | | |
| No | 68/115 (59) | - | - | - | - |
| Yes | 34/52 (65) | 1.3 (0.7, 2.6) | 1.2 (0.6, 2.5) | 1.1 (0.5, 2.4) | 1.2 (0.5, 2.7) |
| Employer provides gloves | | | | | |
| No | 46/81 (57) | - | - | - | - |
| Yes | 56/86 (65) | 1.4 (0.8, 2.7) | 1.3 (0.6, 2.5) | 1.1 (0.5, 2.4) | 1.3 (0.6, 2.9) |

*Note:* OR = odds ratio; CI = confidence interval; aOR = adjusted odds ratio. Sampling date was modelled as continuous (days from WHO COVID-19 pandemic declaration, March 11, 2020 (Ghebreyesus, 2020), to sampling date); industry was modelled as categorical (animal slaughtering and processing, health care and social assistance, animal production and aquaculture, other); age was modelled as categorical (30 or younger, 31 to 40, 41 to 50, 51 to 60, 61 or older); sex was modelled as binary (female or male).

***Table S3.*** *PubMed search strategy for North Carolina SARS-CoV-2 infection-induced seroprevalence estimates.*

| | |
|---|---|
| 1 | (Serology[Mesh] OR “Serologic Tests"[Mesh] OR Immunoglobulins[Mesh] OR “Seroepidemiologic Studies"[Majr] OR sero*[tiab] OR antibody[tiab] OR “anti-body”[tiab] OR antibodies[tiab]) |
| 2 | ("COVID-19"[Mesh] OR "SARS-CoV-2"[Mesh] OR “COVID-19”[tiab] OR coronavirus[tiab] OR “covid 2019”[tiab] OR “SARS-CoV-2”[tiab] OR “severe acute respiratory syndrome coronavirus 2”[tiab]) |
| 3 | "COVID-19 Serological Testing"[Mesh] |
| 4 | ("North Carolina"[Mesh] OR “North Carolina”[tiab] OR “Charlotte”[tiab] OR “Raleigh”[tiab] OR “Greensboro”[tiab] OR “Durham”[tiab] OR “Winson-Salem”[tiab] OR “Fayetteville”[tiab] OR “Cary”[tiab] OR “Wilmington”[tiab]) |
| 5 | (2019/12[Date - Publication]: 3000[Date - Publication]) |
| 6 | ((1 AND 2) OR 3) AND 4 AND 5 |

*Note:* 27 studies identified through PubMed and SeroHub searches, four of which met search criteria (North Carolina-specific infection-induced seroprevalence estimates) (CDC, 2020a, 2020b; Barzin *et al.*, 2020; COVID-19 Community Research Partnership Study Group, 2021). SeroHub is an online dashboard developed by the National Cancer Institute, National Institute of Allergy and Infectious Diseases, and the CDC intended to help researchers and policymakers monitor SARS-CoV-2 seroprevalence studies in the United States (Freedman *et al.*, 2022). Last date of search was October 16, 2023.

***Figure S2.*** *SARS-CoV-2 infection-induced antibody prevalence among animal slaughtering and processing industry workers (n=56) and all employed participants (n=163) in this study compared to North Carolina general population prevalence estimates and PCR+ confirmed COVID-19 cases in North Carolina, 2020-2022.*

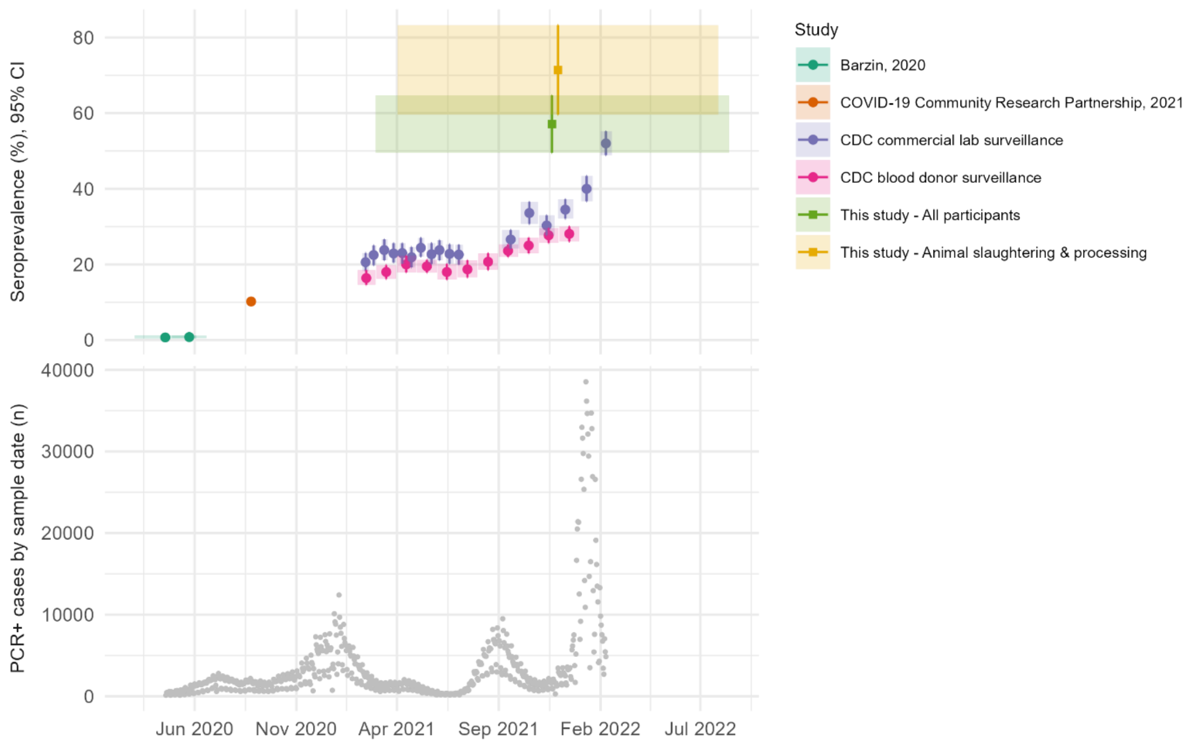


*Note:* See Table S4 for study-specific infection-induced antibody prevalence, study population, and assay information. Barzin, 2020 aimed to quantify asymptomatic spread and therefore these seroprevalence values likely underestimates general population seroprevalence. Horizontal color-shaded area indicates the calendar time period for a given SARS-CoV-2 antibody prevalence estimate. PCR+ confirmed COVID-19 cases in North Carolina, 2020-2022 from North Carolina Department of Health and Human Services COVID-19 dashboard (North Carolina Department of Health and Human Services, 2024).

***Table S4.*** *SARS-CoV-2 infection-induced seroprevalence among the North Carolina general population by midpoint between collection start and end dates.*

| Study | Population description | Assay | Collection dates (year-month-day) | Sample size | Infection-induced seroprevalence, % (95% CI) |
|---|---|---|---|---|---|
| Barzin, 2020 | Inpatients unrelated to COVID-19 | Abbot Architect N | 2020-03-03 to 2020-06-04 | 1449 | 0.7 |
| Barzin, 2020 | Asymptomatic people at NC outpatient clinics | Abbot Architect N | 2020-04-28 to 2020-06-19 | 2973 | 0.8 |
| COVID-19 Community Research Partnership Study Group, 2021 | Wake Forest Baptist Health and Atrium Health systems, general pop | Syntron/Tianjin New Bay Bioresearch N lateral flow assay (LFA) | 2020-04-16 to 2021-01-04 | 11468 | 10.2 |
| CDC commercial lab surveillance | NC commercial lab remnant specimens | Roche Elecsys N | 2021-02-08 to 2021-02-18 | 1309 | 20.6 (18.3,23) |
| CDC blood donor surveillance | Central and Western NC region blood donors | Roche Elecsys N | 2021-02-01 to 2021-02-28 | 4208 | 16.4 (14.6,18.5) |
| CDC commercial lab surveillance | NC commercial lab remnant specimens | Roche Elecsys N | 2021-02-22 to 2021-03-01 | 1172 | 22.5 (19.8,25) |
| CDC commercial lab surveillance | NC commercial lab remnant specimens | Roche Elecsys N | 2021-03-09 to 2021-03-18 | 1140 | 23.8 (21.1,26.6) |
| CDC blood donor surveillance | Central and Western NC region blood donors | Roche Elecsys N | 2021-03-01 to 2021-03-31 | 4072 | 18 (16.2,19.8) |
| CDC commercial lab surveillance | NC commercial lab remnant specimens | Roche Elecsys N | 2021-03-24 to 2021-03-31 | 1362 | 22.9 (20.5,25.6) |
| CDC commercial lab surveillance | NC commercial lab remnant specimens | Roche Elecsys N | 2021-04-05 to 2021-04-14 | 1304 | 23 (20.2,25.6) |
| CDC blood donor surveillance | Central and Western NC region blood donors | Roche Elecsys N | 2021-04-01 to 2021-04-30 | 4195 | 20 (17.9,22.3) |
| CDC commercial lab surveillance | NC commercial lab remnant specimens | Roche Elecsys N | 2021-04-19 to 2021-04-28 | 1303 | 21.9 (19.2,24.6) |
| CDC commercial lab surveillance | NC commercial lab remnant specimens | Roche Elecsys N | 2021-05-03 to 2021-05-12 | 1307 | 24.4 (22.1,27) |
| CDC blood donor surveillance | Central and Western NC region blood donors | Roche Elecsys N | 2021-05-01 to 2021-05-31 | 4159 | 19.5 (17.8,21.2) |
| CDC commercial lab surveillance | NC commercial lab remnant specimens | Roche Elecsys N | 2021-05-17 to 2021-05-29 | 1272 | 22.7 (20.1,25.6) |
| CDC commercial lab surveillance | NC commercial lab remnant specimens | Roche Elecsys N | 2021-05-31 to 2021-06-09 | 1268 | 23.8 (21.1,26.4) |
| CDC blood donor surveillance | Central and Western NC region blood donors | Roche Elecsys N | 2021-06-01 to 2021-06-30 | 4157 | 18 (16,20.1) |
| CDC commercial lab surveillance | NC commercial lab remnant specimens | Roche Elecsys N | 2021-06-14 to 2021-06-25 | 1315 | 22.8 (20.2,25.3) |
| CDC commercial lab surveillance | NC commercial lab remnant specimens | Roche Elecsys N | 2021-06-28 to 2021-07-09 | 1302 | 22.6 (20,25.2) |
| CDC blood donor surveillance | Central and Western NC region blood donors | Roche Elecsys N | 2021-07-01 to 2021-07-31 | 4168 | 18.7 (16.6,21.1) |

| | | | | | |
|---|---|---|---|---|---|
| CDC blood donor surveillance | Central and Western NC region blood donors | Roche Elecsys N | 2021-08-01 to 2021-08-31 | 4226 | 20.7 (18.6,22.9) |
| CDC blood donor surveillance | Central and Western NC region blood donors | VITROS chemiluminescent totat Ig N, Ortho Clinical Diagnostics | 2021-09-01 to 2021-09-30 | 4312 | 23.6 (22,25.3) |
| CDC commercial lab surveillance | NC commercial lab remnant specimens | Roche Elecsys N | 2021-09-06 to 2021-10-02 | 1767 | 26.6 (24.2,29.1) |
| CDC blood donor surveillance | Central and Western NC region blood donors | VITROS chemiluminescent total Ig N, Ortho Clinical Diagnostics | 2021-10-01 to 2021-10-31 | 4330 | 25 (22.9,27.1) |
| CDC commercial lab surveillance | NC commercial lab remnant specimens | Roche Elecsys N | 2021-10-04 to 2021-10-30 | 1775 | 33.6 (30.7,36.5) |
| CDC commercial lab surveillance | NC commercial lab remnant specimens | Roche Elecsys N | 2021-11-01 to 2021-11-24 | 1846 | 30.3 (27.7,33) |
| CDC blood donor surveillance | Central and Western NC region blood donors | VITROS chemiluminescent total Ig N, Ortho Clinical Diagnostics | 2021-11-01 to 2021-11-30 | 4311 | 27.7 (25.7,29.6) |
| CDC commercial lab surveillance | NC commercial lab remnant specimens | Roche Elecsys N | 2021-11-29 to 2021-12-22 | 1833 | 34.5 (32.1,37.2) |
| CDC blood donor surveillance | Central and Western NC region blood donors | VITROS chemiluminescent total Ig N, Ortho Clinical Diagnostics | 2021-12-01 to 2021-12-31 | 4071 | 28.1 (26.1,30) |
| CDC commercial lab surveillance | NC commercial lab remnant specimens | Roche Elecsys N | 2022-01-03 to 2022-01-20 | 1329 | 40 (36.7,43.4) |
| CDC commercial lab surveillance | NC commercial lab remnant specimens | Roche Elecsys N | 2022-02-01 to 2022-02-18 | 1317 | 52 (48.9,55.2) |

*Note:* estimates overlapping with Animal Slaughtering and Processing industry enrollment (April 2, 2021 to July 28, 2022) are highlighted in grey. The minimum prevalence estimate overlapping with Animal Slaughtering and Processing industry enrollment (April 2, 2021 to July 28, 2022) was 18% and maximum 52%. Barzin, 2020 aimed to quantify asymptomatic spread and therefore these seroprevalence values likely underestimates general population seroprevalence.

***Table S5.*** *PubMed search strategy for US animal slaughtering and processing worker SARS-CoV-2 seroprevalence estimates.*

| | |
|---|---|
| 1 | (Serology[Mesh] OR “Serologic Tests"[Mesh] OR Immunoglobulins[Mesh] OR “Seroepidemiologic Studies"[Majr] OR sero*[tiab] OR antibody[tiab] OR “anti-body”[tiab] OR antibodies[tiab]) |
| 2 | ("COVID-19"[Mesh] OR "SARS-CoV-2"[Mesh] OR “COVID-19”[tiab] OR “covid 2019”[tiab] OR “SARS-CoV-2”[tiab] OR “severe acute respiratory syndrome coronavirus 2”[tiab]) |
| 3 | "COVID-19 Serological Testing"[Mesh] |
| 4 | ("Meat-Packing Industry"[Mesh] OR “Poultry”[Mesh] OR “Cattle”[Mesh] OR “Swine”[Mesh] OR “meat pack*”[tiab] OR meatpack*[tiab] OR “meat process*”[tiab] OR slaughterer*[tiab] OR cutter*[tiab] OR meat[tiab] OR poultry[tiab] OR cattle[tiab] OR swine[tiab]) |
| 5 | ("Occupational Groups"[Mesh] OR "Work"[Mesh] OR “Workplace”[Mesh] OR work*[tiab] OR occupation*[tiab] OR industry*[tiab]) |
| 6 | (2019/12[Date - Publication]: 3000[Date - Publication]) |
| 7 | ((1 AND 2) OR 3) AND 4 AND 5 AND 6 |

*Note:* 20 studies identified, only 1 relevant and carried out in the US (Klein *et al.*, 2022). Last date of search was December 14, 2023.

***Table S6.*** *Workplace characteristics and infection prevention and control measures by industry among adult (≥18) employed participants (N=167), North Carolina, 2021-2022.*

| Characteristic, n (%) | Animal slaughtering & processing, N = 57 | Health care & social assistance, N = 20 | Animal production & aquaculture, N = 13 | Other, N = 77 | p-value for any difference |
|---|---|---|---|---|---|
| Essential worker | 56 (98%) | 16 (80%) | 13 (100%) | 39 (52%) | <0.001[a] |
| Worked in person past 2 weeks (at all) | 57 (100%) | 20 (100%) | 13 (100%) | 62 (81%) | <0.001[a] |
| Employees at worksite | | | | | |
| 10 or fewer | 5 (8.8%) | 7 (39%) | 9 (69%) | 22 (31%) | |
| 11-100 | 10 (18%) | 8 (44%) | 3 (23%) | 27 (38%) | |
| 101-1000 | 21 (37%) | 1 (5.6%) | 1 (7.7%) | 20 (28%) | |
| >1000 | 21 (37%) | 2 (11%) | 0 (0%) | 2 (2.8%) | |
| Hours worked per week | | | | | <0.001[a] |
| <40 | 1 (1.8%) | 5 (25%) | 3 (23%) | 26 (34%) | |
| 40 | 17 (30%) | 12 (60%) | 2 (15%) | 21 (27%) | |
| >40 | 39 (68%) | 3 (15%) | 8 (62%) | 30 (39%) | |
| Aware of COVID-19 cases at work past 2 weeks | 49 (86%) | 16 (80%) | 10 (83%) | 68 (88%) | 0.7[a] |
| Able to maintain 6+ feet of distance | 42 (75%) | 12 (60%) | 12 (92%) | 61 (79%) | 0.2[a] |
| Could isolate if COVID-19+ | 51 (96%) | 20 (100%) | 10 (77%) | 70 (93%) | 0.078[a] |
| Could quarantine if COVID-19 exposed | 49 (92%) | 20 (100%) | 11 (85%) | 72 (96%) | 0.2[a] |
| | | | | | |
| *Infection prevention and control measures* | | | | | |
| *Engineering controls* | | | | | |
| Physical barriers between stations | 18 (32%) | 6 (30%) | 2 (15%) | 28 (36%) | 0.5[a] |
| Added hand washing stations | 44 (77%) | 14 (70%) | 9 (69%) | 47 (61%) | 0.3[a] |
| *Administrative controls* | | | | | |
| Change in workplace sick leave | 12 (21%) | 12 (60%) | 1 (7.7%) | 23 (30%) | 0.004[a] |
| COVID-19 testing at work | 29 (51%) | 11 (55%) | 2 (15%) | 16 (21%) | <0.001[a] |
| Masks required | 50 (88%) | 18 (90%) | 7 (54%) | 55 (71%) | 0.011[a] |
| *PPE* | | | | | |
| Employer provides face masks (any type) | 53 (93%) | 18 (90%) | 7 (54%) | 63 (82%) | 0.007[a] |
| Employer provides N95/KN96/respirators | 9 (16%) | 7 (35%) | 4 (31%) | 13 (17%) | 0.2[a] |
| Employer provides surgical masks | 36 (63%) | 16 (80%) | 4 (31%) | 42 (55%) | 0.030[b] |
| Employer provides cloth masks | 28 (49%) | 4 (20%) | 0 (0%) | 33 (43%) | 0.003[b] |
| Employer provides face shields | 28 (49%) | 7 (35%) | 3 (23%) | 14 (18%) | 0.001[a] |
| Employer provides hand protection | 44 (77%) | 12 (60%) | 7 (54%) | 23 (30%) | <0.001[b] |

[a] Fisher's exact test for any difference in characteristic among industry categories

[b] Pearson's Chi-squared test for any difference in characteristic among industry categories

## REFERENCES


Barzin A, Schmitz JL, Rosin S, et al. (2020) SARS-CoV-2 Seroprevalence among a Southern U.S. Population Indicates Limited Asymptomatic Spread under Physical Distancing Measures. *mBio*; **11**: e02426-20. p. e02426-20.

CDC. (2020a) Nationwide COVID-19 Infection-Induced Antibody Seroprevalence (Commercial laboratories). *CDC COVID Data Tracker*. Available at https://covid.cdc.gov/covid-data-tracker/#national-lab. Accessed 5 October 2023.

CDC. (2020b) 2020-2021 Nationwide COVID-19 Infection- and Vaccination-Induced Antibody Seroprevalence (Blood donations). *CDC COVID Data Tracker*. Available at https://covid.cdc.gov/covid-data-tracker/#nationwide-blood-donor-seroprevalence. Accessed 5 October 2023.

COVID-19 Community Research Partnership Study Group. (2021) Duration of SARS-CoV-2 sero-positivity in a large longitudinal sero-surveillance cohort: the COVID-19 Community Research Partnership. *BMC Infect Dis*; **21**: 889. p. 889.

Duarte N, Yanes-Lane M, Arora RK, et al. (2022) Adapting Serosurveys for the SARS-CoV-2 Vaccine Era. *Open Forum Infectious Diseases*; **9**: ofab632. p. ofab632.

Freedman ND, Brown L, Newman LM, et al. (2022) COVID-19 SeroHub, an online repository of SARS-CoV-2 seroprevalence studies in the United States. *Sci Data*; **9**: 727. Nature Publishing Group. p. 727.

Ghebreyesus TA. (2020) WHO Director-General’s opening remarks at the media briefing on COVID-19 - 11 March 2020. Available at https://www.who.int/director-general/speeches/detail/who-director-general-s-opening-remarks-at-the-media-briefing-on-covid-19---11-march-2020. Accessed 5 July 2023.

Klein MD, Sciaudone M, Richardson D, et al. (2022) SARS-CoV-2 seroprevalence and risk factors among meat packing, produce processing, and farm workers. *PLOS Global Public Health*; **2**: e0000619. Public Library of Science. p. e0000619.

North Carolina Department of Health and Human Services. (2024) NC COVID-19 Dashboard Data. Available at https://covid19.ncdhhs.gov/dashboard/data-behind-dashboards. Accessed 1 May 2024.

Pisanic N, Antar AAR, Kruczynski KL, et al. (2023) Methodological approaches to optimize multiplex oral fluid SARS-CoV-2 IgG assay performance and correlation with serologic and neutralizing antibody responses. *J Immunol Methods*; **514**: 113440. p. 113440.
